# Draft Genome Sequence of Streptococcus equinus (Streptococcus bovis) HC5, a Lantibiotic Producer from the Bovine Rumen

**DOI:** 10.1128/genomeA.00085-15

**Published:** 2015-03-05

**Authors:** Analice C. Azevedo, Cláudia B. P. Bento, Jeronimo C. Ruiz, Marisa V. Queiroz, Hilário C. Mantovani

**Affiliations:** a Departamento de Microbiologia, Universidade Federal de Viçosa, Viçosa, Minas Gerais, Brazil; b Fundação Oswaldo Cruz, Instituto Rene Rachaou (IRR), Belo Horizonte, Minas Gerais, Brazil

## Abstract

Streptococcus equinus (Streptococcus bovis) HC5 is a bacteriocinogenic lactic acid bacterium with simple growth requirements. The draft genome sequence of S. equinus HC5 consists of 1,846,241 bp, with a G+C content of 37.04%. *In silico* analysis indicated that S. equinus HC5 might be useful to control bacteria that are detrimental to livestock animals.

## GENOME ANNOUNCEMENT

Streptococcus equinus (Streptococcus bovis) is an amylolytic lactic acid bacterium commonly associated with ruminal acidosis in cattle (1) and gastrointestinal infections in humans (2). S. equinus HC5 produces bovicin HC5, a lantibiotic that shows a broad spectrum of activity and potential applications in food preservation and livestock production (3). Bovicin HC5 interacts with the peptidoglycan precursor lipid II (4) and shows low *in vitro* and *in vivo* toxicity (5).

Whole-genome paired-end sequencing was performed using the Illumina HiSeq 2000 (Illumina, Inc.) with 38,556,214 reads identified comprising 3,894,177,614 bp and an *N*_50_ contig length of 386,285 bp. The nucleotide sequences were assembled into 11 contigs, with an average length of 167,837 bp, using the SOAP*denovo* package version 1.05. The coding DNA sequences (CDSs) were predicted using Glimmer version 3.02. A homologous comparison of all genes was performed using BLAST algorithms within the GenBank database and RAST (6) for function annotation. Putative genes related to bacteriocin biosynthesis were identified by using the BAGEL3 (7) and antiSMASH (8) softwares.

We obtained 8 scaffolds with a total length of 1,846,241 bp and a G+C content of 37.04%. The genome contains one rRNA operon and 34 tRNAs, and an analysis of the functional categories of the genes identified 1,773 protein-coding sequences, with 930 sequences distributed in 24 subsystem categories by RAST, and 338 sequences were assigned as hypothetical proteins.

The starch-fermenting ability of S. equinus HC5 was related with the presence of three α-amylases, two endo-β-1,3-1,4 glucanases, and one pullulanase. The genome analysis revealed 48 genes involved in substrate uptake, which included 21 genes of the phosphotransferase system (PTS) for the transport of glucose, fructose, mannose, trehalose, maltose, sucrose, cellobiose, lactose, and β-glucosides.

Most streptococci are auxotrophic for several amino acids and vitamins, but S. equinus has an absolute requirement only for biotin, while thiamine stimulates growth (9). The genome of S. equinus HC5 revealed 204 genes related to the biosynthetic pathways of the 20 essential amino acids, including the glutamine synthetase (*glnA*) and glutamate synthase genes (*gltAB*) required for the assimilation of ammonia as the sole source of nitrogen.

The lantibiotic gene cluster for bovicin HC5 (*bvcATCBFEGRK*) is 10.1 kb long and shows an organization similar to that of the streptin operon (10). *In silico* analysis demonstrated that S. equinus HC5 also harbors a class II precursor gene similar to bovicin 255. Additionally, homologs of the two-component system *nsuRK* and the immunity genes *nsuFEG* found in the nisin U producer Streptococcus uberis were found in the genome of S. equinus HC5.

The availability of the first draft genome sequence of S. equinus HC5 provides opportunities for biotechnological exploitation of its genome features with regard to the production of a large spectrum bacteriocin with the potential to be used commercially as an alternative to growth promoters in animal husbandry and as an additive in food preservation.

### Nucleotide sequence accession numbers.

This whole-genome shotgun project has been deposited in DDBJ/ENA/GenBank databases under the accession no. JPGC00000000. The version described in this paper is the first version, JPGC00000000.1.
